# Crosslinking-Dependent Design of Hyaluronic Acid Matrices for Enhanced Bioadhesion and Cellular Response

**DOI:** 10.3390/pharmaceutics18050631

**Published:** 2026-05-21

**Authors:** Alina Diana Panainte, Cătălina Anișoara Peptu, Andreea Crețeanu, Nela Bibire, Isabella Nacu, Liliana Vereștiuc, Eliza Grațiela Popa, Larisa Păduraru, Liliana Mititelu Tartau, Radu Dănilă, Tudor Bibire, Catalina Natalia Yilmaz

**Affiliations:** 1 Faculty of Pharmacy, Grigore T. Popa University of Medicine and Pharmacy Iasi, 16 Universitatii Street, 700116 Iasi, Romania; alina-diana.panainte@umfiasi.ro (A.D.P.); nela.bibire@umfiasi.ro (N.B.); eliza.popa@umfiasi.ro (E.G.P.); larisa-paduraru@umfiasi.ro (L.P.); 2 Faculty of Chemical Engineering and Environmental Protection, Gheorghe Asachi Technical University of Iasi, 71 Prof. Dr. Docent Dimitrie Mangeron Street, 700050 Iasi, Romania; catalina-anisoara.peptu@academic.tuiasi.ro; 3 Faculty of Medical Bioengineering, Grigore T. Popa University of Medicine and Pharmacy Iasi, 16 Universitatii Street, 700116 Iasi, Romania; isabella.nacu@umfiasi.ro (I.N.); liliana.verestiuc@umfiasi.ro (L.V.); 4 Faculty of Medicine, Grigore T. Popa University of Medicine and Pharmacy Iasi, 16 Universitatii Street, 700116 Iasi, Romania; liliana.tartau@umfiasi.ro (L.M.T.); radu.danila@umfiasi.ro (R.D.); tudor.bibire@umfiasi.ro (T.B.); 5 Faculty of Science, Dokuz Eylül University, Izmir 35390, Turkey; catalinanatalia.yilmaz@deu.edu.tr

**Keywords:** thiolated hyaluronic acid, hydrogels, polyvinyl alcohol, physical crosslinking, carbodiimide crosslinking, piroxicam, tissue engineering

## Abstract

Hyaluronic acid (HA) hydrogels have attracted increasing interest for biomedical applications due to their tunable properties and biocompatibility. **Methods:** In this study, hyaluronic acid HA-based hydrogels were developed using two distinct crosslinking strategies: physical crosslinking through poly(vinyl alcohol) (PVA) incorporation and covalent crosslinking via DCC/NHS-mediated reactions. Piroxicam (Px) was included as a model drug to evaluate the drug delivery potential of the resulting systems. The hydrogels were characterized in terms of morphology, swelling behaviour, adhesion, enzymatic degradation, drug release, and in vitro cytocompatibility. **Results:** The results indicate that formulation parameters significantly influence the overall performance of the systems. PVA-containing hydrogels exhibited higher swelling capacity and improved adhesive properties, while covalently crosslinked networks showed reduced swelling and enhanced structural stability and resistance to enzymatic degradation. Drug release profiles were dependent on network structure, with more compact systems displaying slower release behaviour. In vitro assays suggested that the developed hydrogels are cytocompatible and that drug incorporation influences both release kinetics and cellular response. However, it should be noted that the biological evaluation was performed under simplified in vitro conditions, which primarily reflect specific aspects such as cell viability and migration. **Conclusions**: This study provides a comparative analysis of physical and covalent crosslinking strategies within a HA platform and highlights how formulation variables influence key physicochemical and biological properties. These findings contribute to the rational design of HA-based hydrogels, although further studies are required to establish their performance in more complex biological environments.

## 1. Introduction

The design and development of advanced biomaterials have become central objectives in modern biomedical engineering [1], where the need for functional, biocompatible, and highly controllable formulations is rapidly increasing. In particular, hydrogels—three-dimensional hydrophilic polymer networks [2] capable of retaining large amounts of water—represent a major class of soft materials with applications ranging from tissue engineering and wound healing to drug delivery and cell encapsulation [3,4,5]. Their structural similarity to native extracellular matrix (ECM), combined with the ability to fine-tune their mechanical properties, degradation profile and biological interactions, make hydrogels ideal candidates for next-generation therapeutic platforms [6]. A critical component of successful formulation is the strategic selection and combination of polymers [7], crosslinkers and activating agents [8] to obtain materials that meet specific functional and biomedical requirements. Accordingly, understanding how chemical structure, cross-linking mechanisms and composite formulations influence hydrogel performance is essential for guiding rational design in biomaterials science [9].

Hyaluronic acid (HA), a naturally occurring glycosaminoglycan, is one of the most widely used biopolymers for hydrogel formulations [10], potentially due to its intrinsic biocompatibility, viscoelasticity, and involvement in key physiological processes such as cell migration, wound repair and tissue hydration [11]. However, the use of unmodified HA is limited by rapid enzymatic degradation and insufficient mechanical stability, which restrict its use in long-term or mechanically demanding applications. To overcome these shortcomings, various strategies have been developed to modify the HA backbone, including covalent cross-linking, grafting of functional groups, and blending with synthetic polymers [12]. Such formulation approaches aim to enhance structural integrity, modulate swelling behaviour, improve adhesion to biological tissues and enable controlled drug release.

Incorporation of thiol-containing molecules such as cysteine (CY) offers a versatile approach for HA functionalization. Cysteine provides both amine and thiol groups that participate in covalent coupling, disulfide formation, and redox-responsive interactions, enabling the construction of hydrogels with dynamic behaviour and tunable mechanical properties [13]. Thiolated HA systems are known to exhibit improved mucoadhesion, higher elasticity, and enhanced cellular compatibility, making them attractive for topical, trans-dermal, and injectable biomedical applications [14,15]. Their ability to form reversible disulfide bonds enables controlled degradation and responsiveness to the physiological environment.

More robust crosslinking can be achieved through carbodiimide chemistry [16], particularly the widely employed N, N′-dicyclohexylcarbodiimide (DCC) in combination with N-hydroxysuccinimide (NHS). The DCC/NHS activation pathway is a commonly employed strategy for promoting amide bond formation between carboxyl and amino groups [17]. This activation strategy enhances coupling efficiency, reduces side reactions, and yields densely crosslinked hydrogels with superior stability and mechanical strength. Such formulations are particularly valuable when the intended application requires slow degradation, high durability, or resistance to mechanical stress.

To further refine hydrogel performance, formulation strategies often incorporate secondary polymers. Poly(vinyl alcohol) (PVA) is an important synthetic polymer, widely used in pharmaceutical and biomedical formulations due to its high hydrophilicity, film-forming ability and capacity to create strong hydrogen-bonded networks [18,19]. The incorporation of PVA with HA-based matrices generates hydrogels with enhanced properties, such as: elasticity, increased swelling capabilities and improved adhesion to wet biological surfaces, attributes essential for wound dressings [20], tissue scaffolds [21] and topical delivery systems [22].

Piroxicam (Px), a nonsteroidal anti-inflammatory drug (NSAID), has attracted considerable interest for localized drug delivery applications due to its anti-inflammatory activity, relatively low molecular weight, and favourable transdermal permeability [23,24,25,26,27]. However, its poor aqueous solubility and the systemic adverse effects associated with prolonged oral administration may limit its therapeutic efficiency [28,29]. Incorporation of Px into hydrogel matrices represents a promising strategy to improve local drug retention, achieve sustained release, and reduce systemic exposure while maintaining therapeutic activity [30,31]. In particular, HA-based hydrogels provide a suitable platform for localized Px delivery because of their high biocompatibility, hydration capacity, and mucoadhesive properties. Previous studies have also suggested the potential usefulness of Px-loaded biomaterials in wound healing and tissue regeneration applications [32,33,34,35]. Nevertheless, most reported studies [36,37,38,39,40,41] have primarily focused on drug delivery performance, while comparatively limited attention has been directed toward understanding how crosslinking strategy and hydrogel architecture influence the physicochemical, adhesive, and biological behaviour of Px-loaded HA systems.

Despite the extensive literature on hyaluronic acid-based hydrogels and their modification through thiolation, polymer blending, or carbodiimide crosslinking, direct comparative studies that systematically evaluate the structure–function relationship remain limited. In particular, the interplay between physical (polymer blending) and covalent (DCC/NHS-mediated) crosslinking, and their combined impact on key functional properties such as swelling, adhesion, degradation, drug release, and biological response, is not yet fully clarified.

Therefore, the present study does not aim to introduce entirely new components, but rather to provide a systematic and comparative evaluation of formulation strategies within a unified thiolated hyaluronic acid system. By integrating PVA blending, carbodiimide-mediated crosslinking, and Piroxicam (Px) incorporation, this work seeks to elucidate how compositional and crosslinking variables influence the resulting physico-chemical and biological performance, thereby contributing to a more rational design of HA-based hydrogels for biomedical applications.

## 2. Materials and Methods

### 2.1. Reagents

Sodium hyaluronate (Mw: 1.5–2.2 × 10^6^ Da) was purchased from Acros Organics (Fair Lawn, NJ, USA), and poly(vinyl alcohol) (PVA) (Mw: approx. 130,000 Da) was obtained from Sigma-Aldrich (Munich, Germany). Piroxicam (Px) (99% purity), used as the model therapeutic agent, was kindly provided by S.C. Antibiotice S.A. (Iasi, Romania). Phosphate buffer solutions (PBS, pH = 7.4) were prepared using sodium phosphate dibasic heptahydrate (Na_2_HPO_4_, ≥99.0%), and potassium phosphate monobasic (KH_2_PO_4_, ≥98.0%), both sourced from Sigma-Aldrich. For the in vitro cytotoxicity assays, human primary fibroblasts (HDFa, cell line, Gibco™ Thermo Fisher Scientific, Waltham, MA, USA) and specific reagents for cell cultures—Dulbecco’s Modified Eagle Medium (DMEM F12 HAM), fetal bovine serum (FBS, sterile-filtered, suitable for cell culture), and an antibiotic cocktail (penicillin–streptomycin–neomycin; P/S/N), 3-(4.5-dimethyl-2-thiazolyl)-2.5-diphenyl-2H-tetrazolium bromide and dimethyl sulfoxide (DMSO, purity ≥99.9%)—were utilized. These biological reagents were purchased from Sigma-Aldrich (Steinheim, Germany). All aqueous solutions were prepared using deionized water (18.2 MΩ·cm).

### 2.2. Methods

#### 2.2.1. Hydrogels Preparation

**Figure 1:**
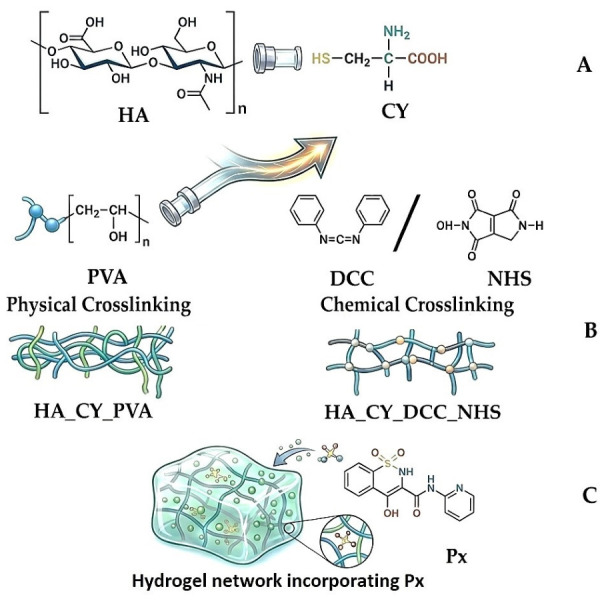
Schematic illustration of (**A**) Main components; (**B**) Hydrogel formation via physical crosslinking with PVA and covalent crosslinking using DCC/NHS chemistry; (**C**) Incorporation of Px within the hydrogel network (HA—hyaluronic acid; PVA—poly(vinyl alcohol); DCC/NHS—N′-dicyclohexylcarbodiimide in combination with N-hydroxysuccinimide; Px—piroxicam). This imagine was generated using Google Gemini.

A schematic illustration of hydrogel preparations is given in Figure 1.

##### Preparation of HA_CY Hydrogel

A concentrated aqueous solution of L-cysteine (0.141 g, 1.16 mmol) was added dropwise to 5 g of a 1% (*w*/*v*) aqueous hyaluronic acid (HA) solution containing approximately 0.05 g HA (corresponding to ~0.125 mmol of HA repeating units). The approximate molar ratio between HA carboxyl groups and cysteine was therefore 1:9. The HA solution was confirmed to be completely clear and homogeneous prior to addition. The resulting mixture was maintained under continuous stirring at room temperature for 4 h. The HA_CY conjugate was subsequently recovered by precipitation in acetone.

##### Preparation of HA_CY_DCC_NHS Conjugate

A 1% (*w*/*v*) hyaluronic acid (HA) solution was prepared by dissolving 0.1 g sodium hyaluronate in 10 mL ultrapure water under continuous stirring until complete homogenization. The pH was adjusted to 5.0 using 0.1 M HCl. Subsequently, N-hydroxysuccinimide (NHS, 0.046 g, 0.40 mmol) and N,N′-dicyclohexylcarbodiimide (DCC, 0.0833 g, 0.40 mmol) were added to activate the carboxyl groups of HA. After 45 min of stirring, an aqueous solution of L-cysteine (0.1405 g, 1.16 mmol) was added dropwise to the activated HA solution. The approximate molar ratio between HA carboxyl groups, DCC, NHS, and cysteine was 1:1.6:1.6:4.7. The reaction mixture was maintained under vigorous stirring for 12 h at room temperature under light-protected conditions and inert atmosphere to minimize thiol oxidation.

##### Preparation of HA_CY_PVA Polymer Blends

A 5% (*w*/*v*) poly(vinyl alcohol) (PVA) solution was added dropwise to a 1% (*w*/*v*) aqueous hyaluronic acid (HA) solution under continuous stirring. The mixture was homogenized for 2 h at room temperature to ensure uniform blending of the polymeric components. Subsequently, an aqueous solution of L-cysteine (0.141 g, 1.16 mmol) was introduced into the polymer blend, corresponding to an approximate HA carboxyl group-to-cysteine molar ratio of 1:9. The reaction mixture was maintained under continuous magnetic stirring at room temperature for 24 h to promote complete interaction between the components and formation of the HA_CY_PVA system.

##### Preparation of Piroxicam-Loaded Hydrogels

For Px-loaded formulations, Piroxicam (Px, 0.7 mg) was first dissolved in a minimal volume of ethanol and subsequently added to the polymeric mixture to ensure homogeneous dispersion of the drug within the hydrogel network.

Both the covalently crosslinked conjugates (HA_CY_DCC_NHS) and the physically crosslinked polymer blends (HA_CY_PVA), in loaded and unloaded forms, were subjected to three successive freeze–thaw cycles to promote network stabilization and physical crosslinking. All formulations were purified by dialysis against acidified ultrapure water for 72 h using 10 kDa molecular weight cut-off dialysis tubing (Thermo Fisher Scientific, MA, USA) to remove residual coupling agents and unreacted cysteine. The dialysis medium was replaced twice daily to maintain the concentration gradient. All procedures were performed under light-protected conditions.

The purified hydrogels were subsequently frozen and lyophilized at −70 °C and 309 mbar for 24 h. The obtained cryogels were stored at 4 °C in a desiccated environment until further characterization. The reaction yield was determined by comparing the initial mass of the reactants with the final mass of the lyophilized hydrogels. Following purification and lyophilization, the physically cross-linked hydrogels (HA_CY and HA_CY_PVA) were recovered with yields exceeding 85%, whereas the covalently cross-linked HA_CY_DCC_NHS formulations showed lower recovery yields of approximately 57%, likely due to purification losses associated with the removal of residual coupling agents and reaction byproducts.

#### 2.2.2. Determination of Thiol Group Content

The thiol content of the HA_CY conjugates was determined spectrophotometrically using Ellman’s reagent (5,5′-dithiobis-(2-nitrobenzoic acid), DTNB; ≥98% purity, Thermo Fisher Scientific, Rockford, IL, USA), according to the colorimetric method described by Li et al. [42]. The assay is based on the reaction between free sulfhydryl groups and DTNB, resulting in the formation of the yellow-coloured 5-thio-2-nitrobenzoic acid (TNB) chromophore. Absorbance measurements were recorded at 412 nm using an Agilent 8453 UV–Vis spectrophotometer (PerkinElmer, Shelton, CT, USA), and the thiol concentration was calculated from a calibration curve prepared using standard L-cysteine solutions. All measurements were performed in triplicate, and the results were expressed as µmol thiol groups per gram of dry hydrogel.

#### 2.2.3. Structural Analysis by ATR-FTIR

The chemical structure of the individual components and the resulting lyophilized hydrogels was characterized using attenuated total reflectance Fourier transform infrared (ATR-FTIR) spectroscopy. Measurements were performed using a PerkinElmer ATR-FTIR spectrometer (Shelton, CT, USA). Prior to analysis, representative hydrogel samples were finely ground and directly applied onto the diamond ATR crystal to ensure optimal spectral acquisition. Spectra were recorded at room temperature over the range of 4000–500 cm^−1^ with a spectral resolution of 4 cm^−1^.

#### 2.2.4. Morphological Analysis

The morphology of the lyophilized hydrogels was investigated by scanning electron microscopy (SEM) using a Hitachi SU1510 microscope (Tokyo, Japan). Prior to analysis, the samples were freeze-dried to preserve their internal porous architecture, mounted onto aluminum stubs, and sputter-coated with a thin gold layer (~7 nm). SEM micrographs were obtained at an accelerating voltage of 5 kV.

#### 2.2.5. Swelling Degree Assay

The swelling behaviour of the freeze-dried hydrogels was evaluated in phosphate-buffered saline (PBS, 0.01 M, pH 7.4) at 37 °C. Samples were placed in QIAquick VR Spin Columns (Ø10 mm) connected to 1 mL syringes and incubated in PBS under static conditions. The swelling capacity was determined at predetermined time intervals until equilibrium swelling was reached after approximately 5 h.

At each time point, the amount of absorbed PBS was determined gravimetrically, and the swelling degree (SD, %) was calculated according to Equation (1):

SD % = (W_t_ − W_0_)/W_0_ × 100 (%), (1)

where W_0_ represents the initial weight of the freeze-dried hydrogel and W_t_ represents the weight of the swollen hydrogel at time t. All experiments were performed in triplicate, and the results are presented as mean ± standard deviation (SD).

#### 2.2.6. Drug Loading (DL) and Encapsulation Efficiency (EE%)

To determine the Piroxicam (Px) content, 0.5 g of the Px-loaded freeze-dried hydrogel was immersed in phosphate-buffered saline (PBS, pH 7.4) at room temperature for 24 h to ensure complete swelling and extraction of Px. The resulting suspension was filtered using 0.2 µm Whatman filters (Maidstone, UK), and the Px concentration was quantified via High-Performance Liquid Chromatography (HPLC) according to the method described by Panainte et al. [43] The drug loading (DL) and entrapment efficiency (EE) percentage were calculated using Equations (2) and (3) [44]:

DL (mg/g) = M_Px_/M_L_, (2)

EE% = (M_Px_/M_i_) × 100, (3)

where MPx is the mass of Px detected in the hydrogel (mg); ML is the total mass of the loaded freeze-dried hydrogel (g); and Mi is the initial mass of Px added during the preparation (mg).

All experiments were conducted in triplicate (n = 3) to ensure reproducibility, and the data are expressed as the mean ± standard deviation (SD).

#### 2.2.7. In Vitro Release Profile of Piroxicam

The release profile of Piroxicam (Px) from the loaded hydrogel matrices was evaluated in phosphate-buffered saline (PBS, pH 7.4) over a 5 h period. Briefly, 0.05 g of freeze-dried Px-loaded hydrogel was enclosed in dialysis tubing (molecular weight cut-off 12–14 kDa) and immersed in 50 mL PBS. The system was maintained at 37 °C under constant agitation at 50 rpm to simulate physiological conditions. At predetermined time intervals, 500 μL aliquots were withdrawn from the release medium and immediately replaced with an equal volume of fresh pre-warmed PBS to maintain sink conditions. The collected samples were filtered through 0.22 μm Whatman membranes and analyzed by high-performance liquid chromatography (HPLC) using an injection volume of 20 μL to quantify the released Px.

All experiments were performed in triplicate (n = 3), and the results were expressed as cumulative drug release percentages over time [45]. The cumulative percentage of Px released from the HA_CY_Px, HA_CY_PVA_Px, and HA_CY_DCC_NHS_Px hydrogels was calculated according to Equation (4):

Cumulative Release (%) = C_t_/C_0_ × 100, (4)

where C_t_ = the concentration of Px released at a specific time and C_0_ = the total initial concentration of Px loaded into the hydrogel matrix.

#### 2.2.8. Enzymatic Degradation Assay

The enzymatic degradation behaviour of unloaded and Piroxicam-loaded (Px-loaded) hydrogels was evaluated using a hyaluronidase-mediated biodegradation assay designed to simulate the physiological degradation of the hyaluronic acid matrix. Phosphate-buffered saline (PBS, pH 7.4) was supplemented with bovine testicular hyaluronidase (Type I-S) to obtain a final enzymatic activity of 30 U/mL. Standardized amounts of each hydrogel formulation, including native HA (control), unloaded hydrogels, and Px-loaded hydrogels, were weighted and immersed in 2 mL of enzymatic solution in sterile vials. The samples were incubated at 37 °C under continuous agitation at 50 rpm using a thermostatic orbital shaker.

At predetermined time intervals, the samples were centrifuged at 5000 rpm for 2 min to facilitate removal of the supernatant. The remaining hydrogel mass was subsequently recorded gravimetrically. The weight loss (WL%) during enzymatic degradation was calculated according to the method described by Hutomo et al. [46] using Equation (5):

WL (%) = (W_i_ − W_t_)/W_i_ × 100, (5)

where W_i_ represents as the initial weight of the lyophilized hydrogels and W_t_ represents remaining hydrogel weight after the degradation test.

All experiments were performed in triplicate (n = 3). Data are presented as mean ± standard deviation (SD).

#### 2.2.9. In Vitro Bioadhesion Tests

The bioadhesive properties of the hydrogels were evaluated using a TA.XT Plus^®^ texture analyzer (Stable Micro Systems, Godalming, UK). A cellulose membrane (Sterlitech, Kent, WA, USA; 4 cm^2^, 12,000 Da), commonly used as a tissue-mimetic substrate, was employed for the analysis according to previously reported methods [47,48,49].

Prior to testing, the cellulose membrane was boiled and cooled to room temperature. To simulate physiological conditions, 200 μL of phosphate-buffered saline (PBS, pH 7.4, 0.01 M) was added to the membrane support device. The system was maintained at 37 °C under gentle agitation (200 rpm) throughout the experiment.

Lyophilized hydrogels (ϕ = 8 mm) were attached to the lower surface of a cylindrical graphite probe (P/8, 8 mm diameter). The probe was lowered at a constant speed of 1 mm/s until contact with the membrane and maintained in contact for 30 s under a force of 9.8 mN. The work of adhesion and maximum detachment force were calculated from the obtained force–time curves using Texture Exponent software (version 32).

Five independent replicates were performed for each formulation.

#### 2.2.10. In Vitro Cytocompatibility Assays

Human dermal fibroblast cells (HDFa cell line) were initially cultured in complete Dulbecco’s Modified Eagle Medium (DMEM F12 HAM) supplemented with 10% fetal bovine serum (FBS) and 1% antibiotic mixture (penicillin, streptomycin, and neomycin, P/S/N). Cells were incubated for 24 h under standard conditions: 5% CO_2_, 37 °C, and 95% relative humidity. For the cytocompatibility assay, 48-well plates were seeded with 12 × 10^3^ cells per well from the trypsinized suspension and allowed to adhere for 24 h under the same incubation conditions.

Meanwhile, the freeze-dried hydrogels were sterilized by UV irradiation for 30 min on each side, and then equilibrated in complete DMEM F12 HAM medium at 37 °C for 24 h to reach their swelling equilibrium.

For the cell culture experiment, the swollen hydrogels were added to the adhered cells and incubated for 72 h under standard conditions (5% CO_2_, 37 °C, 95% relative humidity). Control wells containing cells without any material were also prepared.

##### MTT Assay

Cell viability was evaluated using the MTT assay according to previously reported methods [50]. Prior to analysis, the hydrogels were removed from each well, and the culture medium was replaced with a 5% MTT working solution prepared in DMEM F12 HAM medium. Cells were incubated with the MTT solution for 3 h at 37 °C under standard culture conditions to allow the formation of formazan crystals.

Subsequently, the formed formazan crystals were dissolved using 500 μL/well dimethyl sulfoxide (DMSO), and the absorbance of the resulting solution was measured at 570 nm using a Tecan Sunrise microplate reader (Tecan Trading AG, Männedorf, Switzerland).

Cell viability was calculated by comparing the absorbance values of experimental wells with those of the control wells (cells without material exposure) using Equation (6):

Cell viability = A_sample_/A_control_ × 100, (6)

where A_sample_ represents the absorbance of cells cultured in the presence of hydrogels, while A_control_ represents the absorbance of untreated control cells.

The MTT assay was performed at 24, 48, and 72 h in triplicate. In addition, cell morphology and density were evaluated using an inverted phase-contrast microscope (Leica, Wetzlar, Germany), and representative images were acquired using a 10× objective lens.

##### Live/Dead Cell Staining

A live/dead staining assay was performed to evaluate cell morphology and viability following hydrogel exposure. HDFa cells were cultured with the hydrogel samples under the same conditions described above in 48-well plates at a density of 12 × 10^3^ cells per well for 72 h.

To visualize viable cells, a Calcein AM staining solution (2 μL/mL in HBSS containing Ca^2+^ and Mg^2+^) was added to the wells and incubated for 30 min at 37 °C. Fluorescence images were acquired using a Leica DM IL LED inverted microscope (Leica, Wetzlar, Germany) equipped with phase-contrast and fluorescence filters using a 10× objective lens.

#### 2.2.11. Scratch Assay

The therapeutic potential of various hydrogel formulations and Piroxicam (Px) treatments on the migratory capacity of human dermal fibroblasts (HDFa) was evaluated using a standardized scratch assay [51]. Cells were cultured in DMEM supplemented with 10% FBS and seeded into 24-well plates, followed by a 48 h incubation period to obtain a homogeneous confluent monolayer. A scratch wound (gap) was mechanically induced in the cell monolayer using a sterile 200 μL micropipette tip. Following the injury, the denuded area was exposed to the following experimental groups:

Control—DMEM supplemented with 10% FBS.

Free Drug—Piroxicam (Px) solution.

Hydrogel Matrices—HA_CY, HA_CY_PVA, and HA_CY_DCC_NHS, evaluated in both unloaded and Piroxicam-loaded (Px) states.

The progression of gap closure was monitored via inverted optical microscopy and quantified using ImageJ software (v1.54). The dynamics of wound closure were recorded at specific incubation intervals: 0, 12, 48, and 72 h. The wound closure efficiency (%) was calculated for each triplicate (n = 3) according to Equation (7):

% Wound Closure = A_0_ − A_t_/A_0_ × 100, (7)

where A_0_ = the initial wound area t = 0 and A_t_ = the wound area at time t after the scratch was performed.

### 2.3. Statistical Analysis

The results are presented as mean ± standard deviation (SD). Statistical analysis was performed using one-way analysis of variance (one-way ANOVA), and differences were considered statistically significant at *p* < 0.05.

### 2.4. GenAI Tool Used

Image resolution enhancement was performed using the Gemini 3 Flash multimodal model to reconstruct high-fidelity details and improve visual clarity.

## 3. Results

### 3.1. Synthesis of Unloaded/Loaded with Px Hydrogels

A series of thiolated hyaluronic acid (HA_Cy) derivatives was successfully prepared using physical and covalent crosslinking strategies. Ellman’s spectrophotometric assay confirmed the presence of free thiol groups in all formulations, with thiol contents of 313 µmol/g for HA_CY_DCC_NHS, 233 µmol/g for HA_CY, and 226 µmol/g for HA_CY_PVA. The higher thiol content observed for the chemically conjugated HA_CY_DCC_NHS formulation suggests a more efficient incorporation of cysteine through carbodiimide-mediated coupling.

### 3.2. ATR-FTIR Spectroscopy Analysis

**Figure 2:**
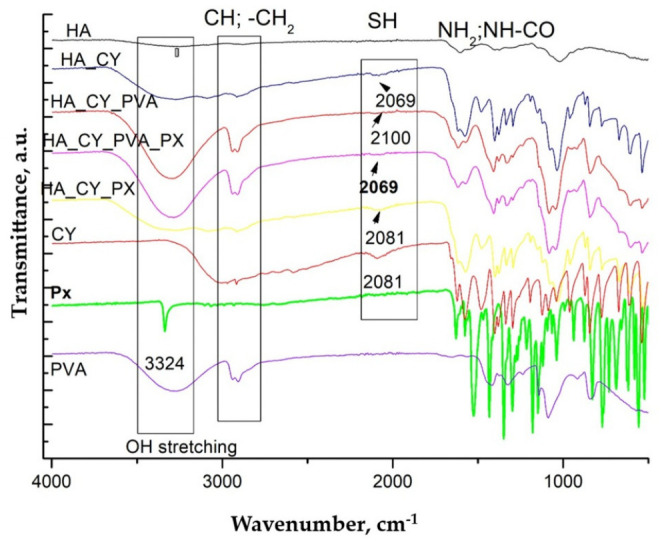
ATR-FTIR spectra of chemical components and of unloaded and Px loaded hydrogels prepared via physical blending.

ATR-FTIR spectroscopy was performed to investigate the chemical structure of the individual components and evaluate the formation of the hydrogel matrices as well as the incorporation of Piroxicam (Px). The spectra of L-cysteine, native HA, PVA, Px, and the hydrogels prepared via physical blending are presented in Figure 2.

The FTIR spectrum of cysteine exhibited characteristic NH_3_+ and CH stretching vibrations within the 3000–2500 cm^−1^ region, together with a weak thiol-associated band near 2081 cm^−1^. In addition, characteristic carboxylate- and amine-related vibrations together with a defined fingerprint region below 1000 cm^−1^ were observed, consistent with previously reported spectra [52,53].

Native HA exhibited characteristic broad hydroxyl and N–H stretching vibrations around 3400 cm^−1^, together with carboxylate- and amide-related bands between 1610 and 1410 cm^−1^. A prominent glycosidic C–O–C stretching vibration was also observed near 1040 cm^−1^, in agreement with previous reports [54,55].

The PVA spectrum presented characteristic hydroxyl stretching vibrations around 3300 cm^−1^, together with CH_2_ stretching bands at 2940 and 2910 cm^−1^ and a strong C–O stretching vibration near 1090 cm^−1^, consistent with previously reported spectra [56,57].

The Px spectrum displayed characteristic N–H stretching vibrations around 3300 cm^−1^ together with amide carbonyl- and aromatic-related bands near 1630 and 1530 cm^−1^ [58]. In addition, sulfonyl-associated vibrations were observed within the 1300–1100 cm^−1^ region [59].

The spectra of HA_CY and HA_CY_PVA hydrogels, both unloaded and Px-loaded, showed broadening of the hydroxyl stretching region between 3000 and 3500 cm^−1^ together with slight shifts in thiol-associated vibrations. In addition, partial overlapping of carboxylate- and amine-related bands within the 1500–1700 cm^−1^ region was observed, suggesting intermolecular interactions between HA, cysteine, PVA, and Px within the hydrogel matrix. The incorporation of Px was further supported by the presence of characteristic aromatic- and sulfonyl-related bands within the 1150–1600 cm^−1^ region.

**Figure 3:**
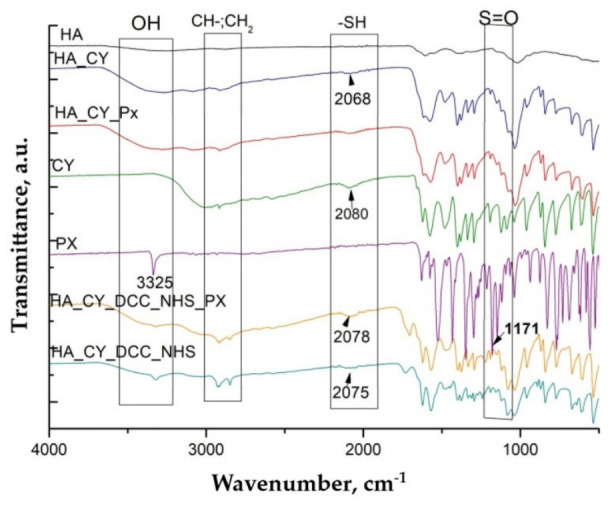
ATR-FTIR spectra of modified HA–Cysteine hydrogels prepared by chemical conjugation and their structural variations.

The FTIR spectra of modified HA hydrogels, prepared via chemical conjugation, are presented in Figure 3.

For the HA_CY_DCC_NHS and HA_CY_DCC_NHS_Px formulations, attenuation and shifting of thiol- and ammonium-related vibrations together with the appearance of more pronounced amide-associated bands within the 1650–1550 cm^−1^ region suggest the formation of covalent interactions between HA and cysteine. Moreover, the persistence of characteristic Px-related bands within the loaded hydrogel spectra indicates successful drug incorporation without evident chemical degradation of the therapeutic agent.

### 3.3. SEM Analysis

**Figure 4:**
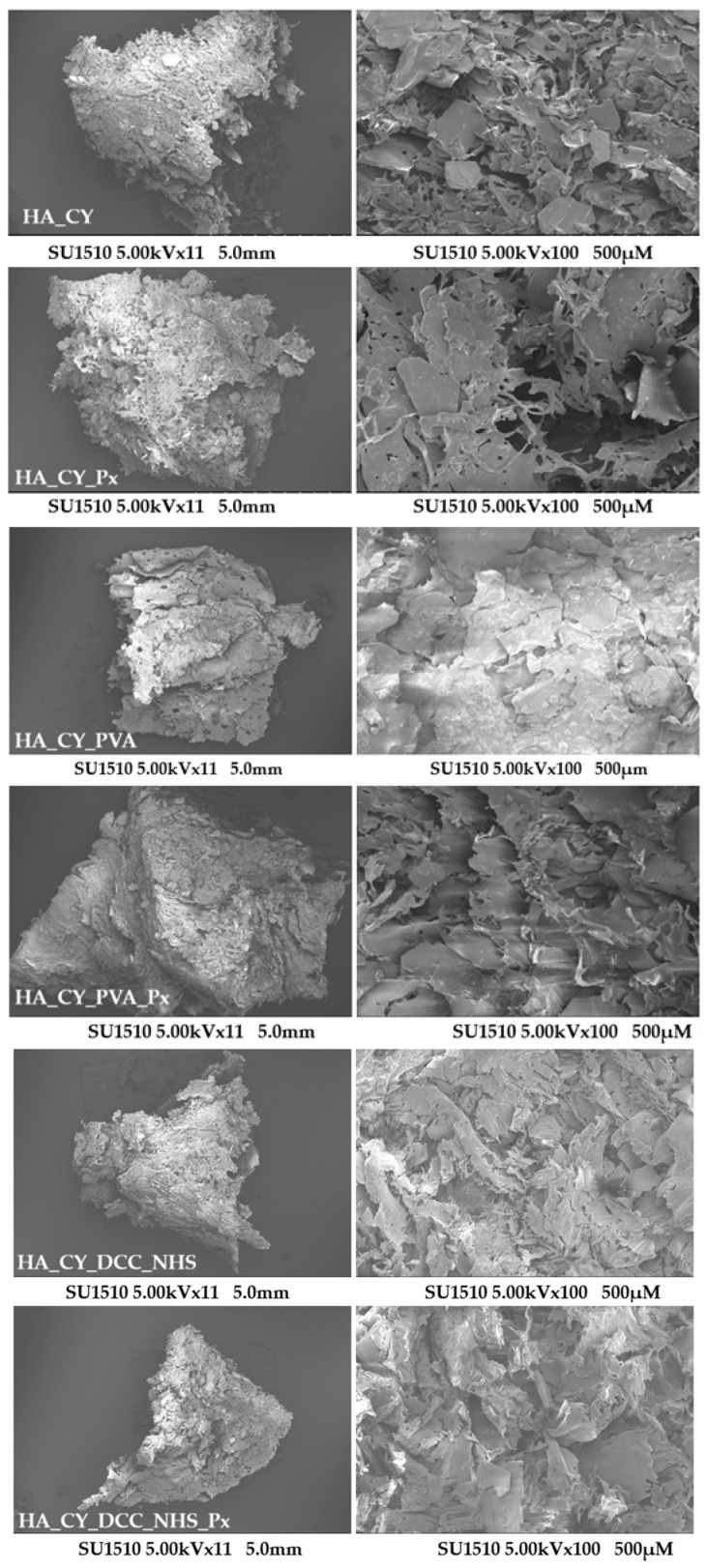
SEM micrographs of lyophilized hydrogels at different magnifications (×11 to ×550). Rows represent different formulations (HA_CY, HA_CY_PVA, HA_CY_DCC_NHS with and without Piroxicam (Px).

Scanning Electron Microscopy (SEM) was used to investigate the morphology of the hydrogels. The SEM micrographs (Figure 4) revealed interconnected lamellar structures with visible microporous regions, forming a heterogeneous network architecture that remained generally preserved after Piroxicam (Px) incorporation.

Differences in morphology were observed between formulations, with the simpler HA_CY systems exhibiting more porous structures, while the PVA-containing and chemically conjugated hydrogels presented denser and more compact morphologies.

The HA_CY formulation exhibited a highly porous and interconnected morphology characterized by thin lamellar structures and open pores. Following Px incorporation, the HA_CY_Px formulation generally retained its porous architecture, although slight thickening of the polymer walls and partial pore narrowing were observed.

The incorporation of PVA in the HA_CY_PVA formulation resulted in a denser and more compact morphology compared to the highly porous HA_CY system. SEM images revealed stacked lamellar domains and a noticeable reduction in pore size and pore interconnectivity. After Px incorporation, the HA_CY_PVA_Px formulation exhibited a further reduction in visible porosity together with a more compact surface morphology, suggesting increased structural densification of the polymer matrix.

The HA_CY_DCC_NHS hydrogels presented a denser and more compact morphology compared to the physically crosslinked formulations, with smaller and less defined porous regions. The chemically conjugated matrices exhibited irregular layered structures and reduced pore interconnectivity, consistent with the formation of a tighter polymer network. Following Px incorporation, the HA_CY_DCC_NHS_Px formulation maintained a highly compact morphology with partial occlusion of the porous architecture.

A gradual transition from highly porous architectures toward more compact layered morphologies was observed following PVA incorporation and chemical conjugation. Compared with HA_CY, these formulations presented smaller and less defined porous regions together with reduced apparent pore interconnectivity.

### 3.4. Swelling Assay Results

**Figure 5:**
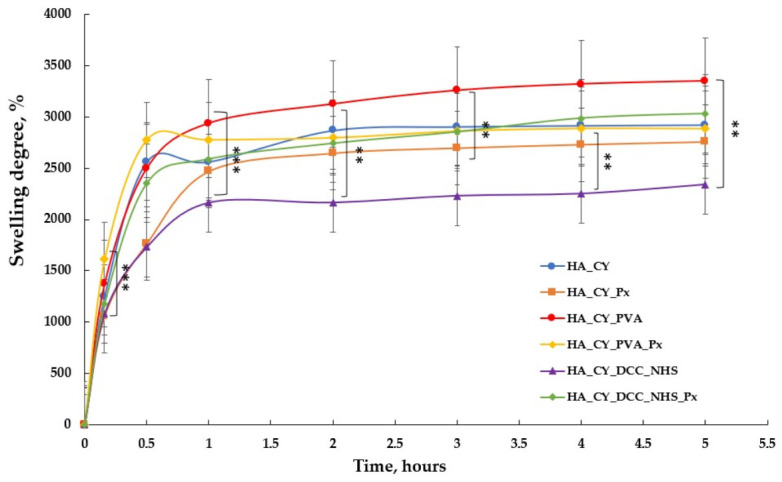
Swelling degree of lyophilized unloaded/loaded hydrogels in phosphate buffer pH = 7.4 at 37 °C. The data are expressed as mean ± standard deviation (SD) with n = 3. ** *p* < 0.01, *** *p* < 0.001.

Figure 5 illustrates the swelling behaviour of the unloaded and Px-loaded hydrogels in PBS (pH 7.4) at 37 °C. Swelling analysis provides important information regarding the hydrophilicity, structural organization, and crosslinking characteristics of hydrogel systems [60].

All hydrogel formulations exhibited a rapid increase in swelling degree during the initial 20–50 min, followed by a gradual stabilization phase until equilibrium swelling was reached after approximately 5 h. This behaviour reflects rapid PBS diffusion into the polymeric matrix followed by equilibration of the hydrated network structure [61,62,63].

Among the investigated formulations, HA_CY_DCC_NHS exhibited the lowest equilibrium swelling degree (~2340%), indicating a comparatively denser network organization. Interestingly, Px incorporation into HA_CY_DCC_NHS_Px increased the swelling degree to approximately 3030%, suggesting partial modification of the hydration behaviour of the polymer matrix.

Overall, the observed swelling differences were closely associated with the composition and crosslinking strategy of the hydrogels. Formulations containing higher proportions of hydrophilic functional groups exhibited enhanced water uptake, whereas more densely crosslinked matrices showed reduced swelling capacity. These findings suggest that hydrogel hydration behaviour can be modulated according to the intended biomedical application.

The swelling study was terminated after 5 h, as all formulations had reached equilibrium swelling, evidenced by minimal changes in PBS uptake between 4 and 5 h [64,65].

Collectively, the swelling results demonstrate the strong influence of polymer composition and crosslinking strategy on the hydration properties of the developed hydrogel systems.

### 3.5. Evaluation of Piroxicam Loading Capacity and Encapsulation Efficiency

**Figure 6:**
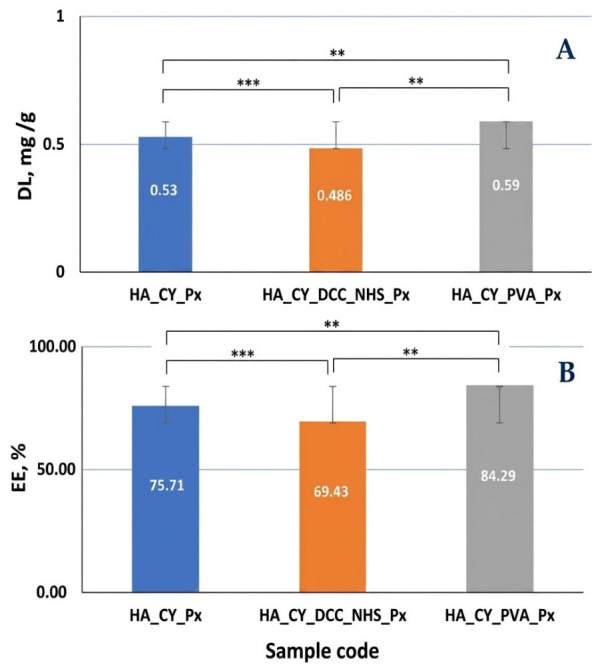
Drug loading (**A**) and entrapment efficiency (**B**). Data are mean ± SD, n = 3. ** *p* < 0.01, *** *p* < 0.001.

The encapsulation efficiency (EE%) of the obtained hydrogels ranged from 69.43% to 84.29% (Figure 6). Differences in drug loading and encapsulation efficiency were associated with the composition of the hydrogels and the applied crosslinking strategy, which influenced the incorporation and retention of Px within the polymeric matrix [58].

The HA_CY_PVA_Px formulation exhibited the highest encapsulation efficiency (84.29%) together with the highest drug loading value (0.59 mg/g) (Figure 6). The im-proved Px retention may be associated with the denser morphology and reduced apparent porosity observed for the PVA-containing matrices, which could contribute to reduced drug loss during hydrogel preparation.

In contrast, the HA_CY_DCC_NHS_Px formulation showed the lowest encapsulation efficiency (69.43%). This behaviour may be related to the more compact morphology and higher crosslinking density of the chemically conjugated matrix, which may have limited the incorporation capacity of the hydrogel network.

The HA_CY_Px formulation presented intermediate encapsulation efficiency values (75.71%), which may be associated with its comparatively open and porous morphology. The higher apparent porosity of this system could facilitate both drug incorporation and partial drug diffusion during processing.

### 3.6. In Vitro Drug Release Study

**Figure 7:**
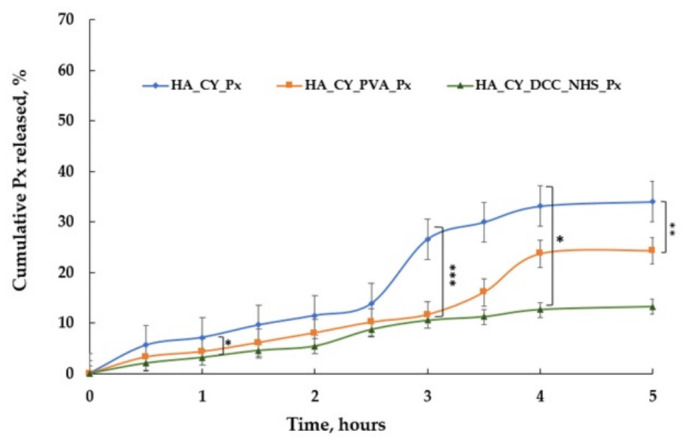
Cumulative % release of Px from HA_CY_Px, HA_CY_PVA-Px, and HA_CY_DCC_NHS_Px. Data are mean ± SD, n = 3. * *p* < 0.05, ** *p* < 0.01, *** *p* < 0.001.

The in vitro release profile of Px from the hydrogel formulations was evaluated in PBS (pH 7.4), and the cumulative percentage of drug released over time is presented in Figure 7.

The modification of the HA matrix significantly influenced the Px release behaviour [66]. The HA_CY_Px formulation exhibited the fastest and highest cumulative release, reaching approximately 40% after 5 h. In contrast, the incorporation of PVA in HA_CY_PVA_Px moderately reduced the release rate, with cumulative release values reaching approximately 30% over the same period. The HA_CY_DCC_NHS_Px formulation exhibited the slowest release profile, with cumulative release remaining below 20% after 5 h.

The slower release observed for the PVA-containing and chemically conjugated formulations may be associated with the formation of denser polymeric networks that restricted Px diffusion from the hydrogel matrix. In addition, the release behaviour appears to be partially swelling-controlled, as formulations exhibiting higher swelling capacity also demonstrated enhanced cumulative drug release. In particular, the DCC/NHS-crosslinked system demonstrated significantly reduced drug release compared with both HA_CY_Px and HA_CY_PVA_Px formulations.

### 3.7. Bioadhesion Results

**Figure 8:**
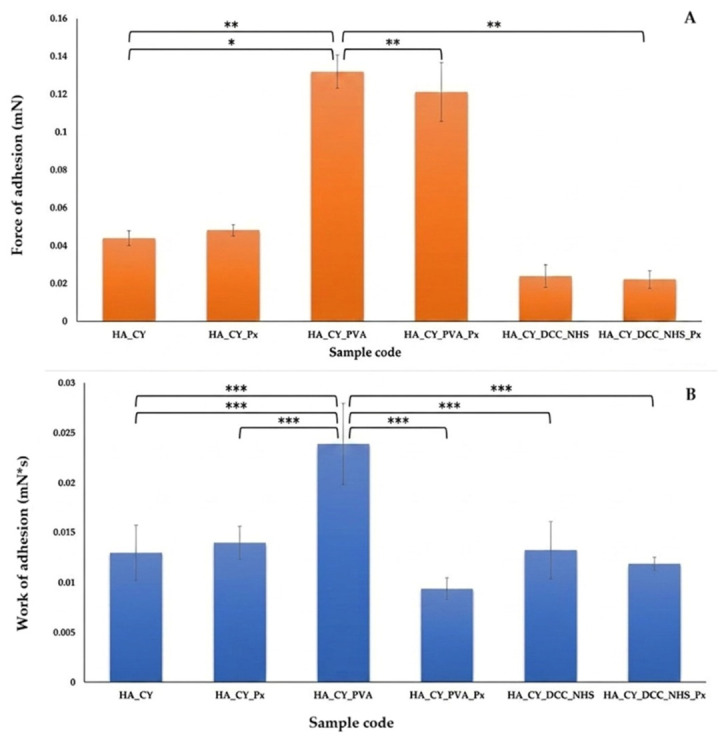
Bioadhesive properties, evaluated as force of adhesion (**A**) and work of adhesion (**B**) of HA_CY-based hydrogels: HA_CY_Px, HA_CY_PVA, HA_CY_PVA-Px, HA_CY_DCC_NHS, and HA_CY_DCC_NHS_Px. Data are mean ± SD, n = 3. * *p* < 0.05, ** *p* < 0.01, *** *p* < 0.001.

The bioadhesive properties of the hydrogels were evaluated by measuring the force of adhesion and the work of adhesion, as presented in Figure 8.

The force of adhesion represents the maximum detachment force required to separate the hydrogel from the substrate, whereas the work of adhesion reflects the overall energy involved in the adhesion and detachment process [67,68].

Among the investigated formulations, HA_CY_PVA and HA_CY_PVA_Px exhibited the highest adhesion forces (~0.12–0.15 mN), indicating enhanced adhesive behaviour compared to the remaining systems. In contrast, HA_CY and HA_CY_Px showed moderate adhesion values (~0.04–0.05 mN), while the DCC/NHS-crosslinked hydrogels exhibited comparatively lower adhesive performance.

The improved adhesion observed for the PVA-containing systems may be associated with the presence of hydrophilic hydroxyl groups together with increased network flexibility and hydration, which can enhance interfacial interactions with the substrate. Conversely, the lower adhesion of the DCC/NHS-crosslinked formulations may be related to the formation of more compact and less flexible polymer networks [69,70,71].

A similar trend was observed for the work of adhesion. HA_CY_PVA exhibited the highest values (~0.025 mN·s), indicating prolonged adhesive interaction with the substrate, whereas the remaining formulations showed lower adhesion energy values. Px incorporation slightly reduced the work of adhesion of the PVA-containing system, possibly due to increased structural compactness of the hydrogel matrix.

Overall, the PVA-containing formulations demonstrated enhanced bioadhesive properties compared with the other hydrogel systems, suggesting their potential suitability for applications requiring prolonged surface contact.

### 3.8. Citocompatibility

**Figure 9:**
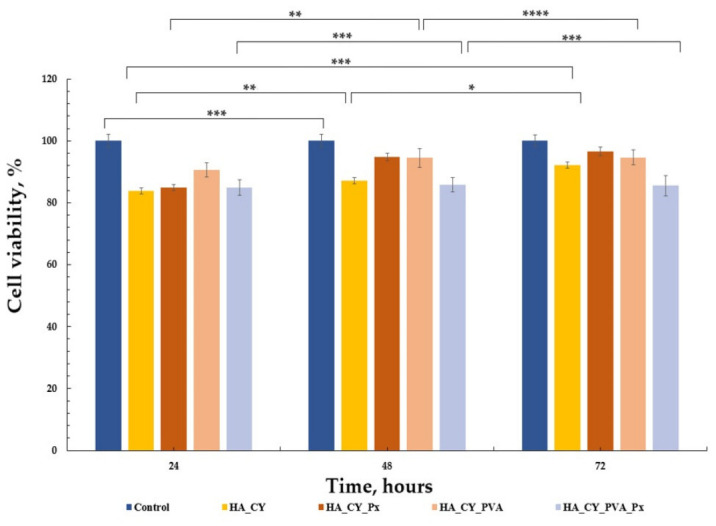
Cell viability of control, HA_CY, HA_CY_Px, HA_CY_PVA and HA_CY_PVA_Px samples after 24, 48, and 72 h of incubation. Data are mean ± SD. * *p* < 0.05, ** *p* < 0.01, *** *p* < 0.001, **** *p* < 0.0001; n = 3.

**Figure 10:**
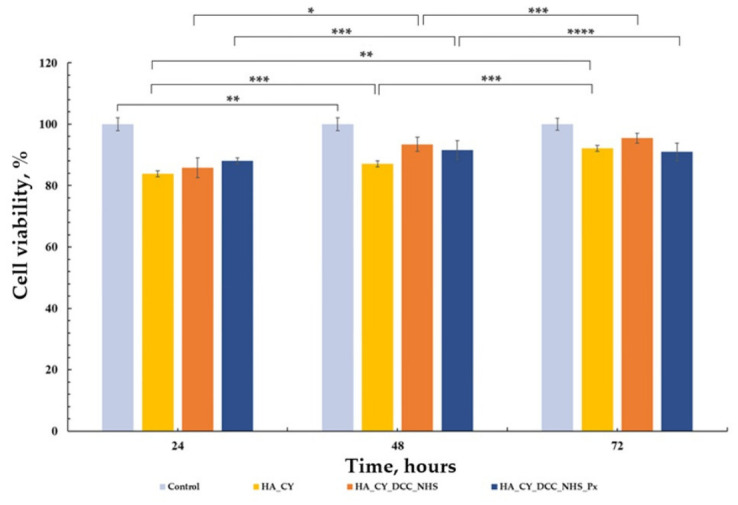
Cell viability of control, HA_CY, HA_CY_DCC_NHS, and HA_CY_DCC_Px samples after 24, 48, and 72 h of incubation. Data are mean ± SD.* *p* < 0.05, ** *p* < 0.01, *** *p* < 0.001, **** *p* < 0.0001; n = 3.

**Figure 11:**
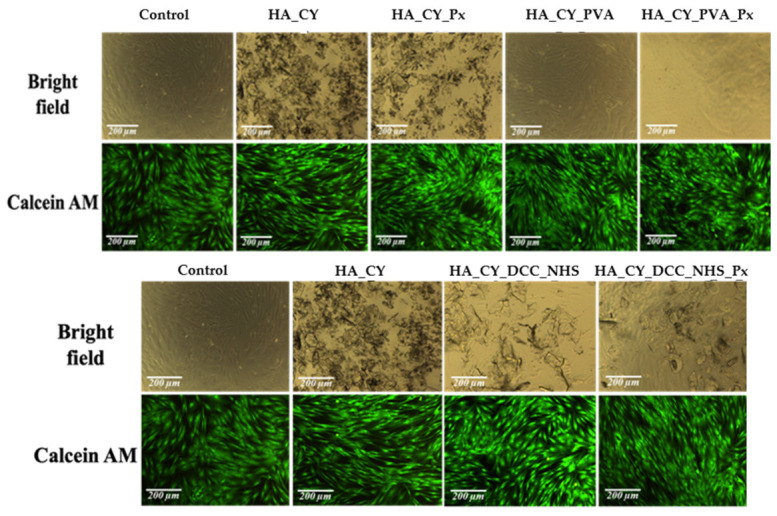
Bright-field (**top row**) and fluorescence (**bottom row**, Live/Dead staining) micrographs of cells cultured on Control, HA_CY, HA_CY_Px, HA_CY_PVA, HA_CY_PVA_Px, HA_CY_DCC_NHS, and HA_CY_DCC_NHS_Px samples after 72 h of incubation.

Following physicochemical characterization, the cytocompatibility of the developed HA_CY-based hydrogels was evaluated using normal human dermal fibroblasts (HDFa cell line). Cell viability was assessed by the MTT assay according to ISO 10993-5 recommendations using a direct-contact approach [72,73,74]. The obtained results are presented in Figure 9, Figure 10 and Figure 11.

The results presented in Figure 9 demonstrate that all HA_CY-based hydrogel formulations maintained high cell viability throughout the 72 h incubation period, indicating favourable cytocompatibility and the absence of significant cytotoxic effects. Cell viability values generally remained above 80% for all investigated samples, confirming that the developed hydrogels support fibroblast survival under direct-contact conditions.

After 24 h of incubation, the HA_CY, HA_CY_Px, and HA_CY_PVA formulations exhibited viability values of approximately 85–90%, while HA_CY_PVA_Px showed slightly lower viability (~80%). This initial reduction may be associated with the comparatively denser structure of the Px-loaded PVA-containing matrix, which could temporarily influence early cell attachment and spreading. However, after 48 and 72 h, all formulations maintained stable viability profiles, suggesting sustained cellular compatibility over time. Among the investigated samples, HA_CY and HA_CY_Px exhibited the highest viability values (>90%), whereas HA_CY_PVA_Px maintained slightly lower but still acceptable viability levels [75].

Overall, the obtained results indicate that both unloaded and Px-loaded HA_CY-based hydrogels exhibit good in vitro cytocompatibility and are capable of supporting fibroblast viability under the investigated experimental conditions.

The cell viability results presented in Figure 10 demonstrate that the HA_CY_DCC_NHS-based hydrogels maintained favourable cytocompatibility throughout the incubation period. The control group exhibited viability values close to 100% at all investigated time points, indicating normal fibroblast growth and proliferation. The HA_CY, HA_CY_DCC_NHS, and HA_CY_DCC_NHS_Px formulations showed slightly lower but comparable viability values, generally ranging between 85% and 95% after 24, 48, and 72 h of incubation.

No significant reduction in cell viability was observed over time, suggesting that the chemically conjugated hydrogels did not induce detectable cytotoxic effects under the investigated conditions. Overall, these findings indicate good in vitro cytocompatibility of the HA_CY_DCC_NHS-based formulations toward human dermal fibroblasts.

The microscopy images presented in Figure 11 illustrate the morphology and viability of fibroblasts cultured on the control and HA_CY-based hydrogels after 72 h of incubation. Bright-field micrographs revealed well-spread and uniformly distributed fibroblast-like cells across all investigated formulations, comparable to the control group. The preservation of the characteristic elongated morphology suggests that the hydrogels did not induce detectable morphological alterations or cytotoxic effects [76].

Live/Dead fluorescence staining further supported these observations. A predominance of green fluorescence corresponding to viable cells was observed for all formulations, whereas only minimal red fluorescence associated with dead cells was detected. In addition, dense and confluent cell layers were visible on several modified HA_CY formulations, indicating favourable conditions for fibroblast attachment and survival.

Overall, the microscopy evaluation was consistent with the quantitative MTT results, confirming good in vitro cytocompatibility of the developed HA_CY-based hydrogels under the investigated experimental conditions.

### 3.9. Evaluation of In Vitro Enzymatic Degradation

**Figure 12:**
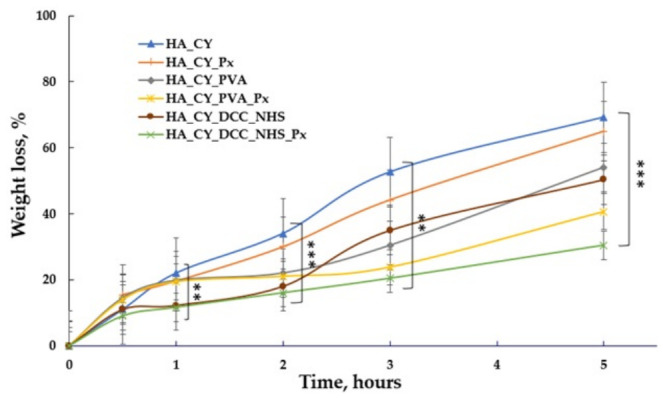
Enzymatic degradation profile for hydrogels loaded/unloaded with Px. Data are mean ± SD, n = 3. ** *p* < 0.01, *** *p* < 0.001.

The enzymatic degradation profiles of the unloaded and Px-loaded hydrogels are presented in Figure 12. The results demonstrate that both the crosslinking strategy and Px incorporation significantly influenced the stability of the HA-based matrices under hyaluronidase exposure.

The HA_CY formulation exhibited the highest degradation degree, reaching approximately 80% after 5 h, indicating limited resistance of the physically stabilized matrix toward enzymatic degradation. In contrast, the chemically conjugated HA_CY_DCC_NHS and HA_CY_DCC_NHS_Px hydrogels showed lower degradation values (~40.6% and 51%, respectively), suggesting improved enzymatic stability associated with covalent crosslinking of the polymer network [69].

The HA_CY_PVA formulation also demonstrated reduced degradation compared with HA_CY, indicating that PVA incorporation contributed to enhanced matrix stability, possibly by reducing enzyme accessibility within the hydrated network. In particular, the HA_CY_PVA_Px formulation exhibited the lowest degradation degree (~30%) after 5 h, indicating enhanced resistance to enzymatic degradation.

Overall, the obtained results suggest that both polymer composition and crosslinking strategy strongly influence the enzymatic stability of the developed hydrogels.

### 3.10. In Vitro Scratch Assay

**Figure 13:**
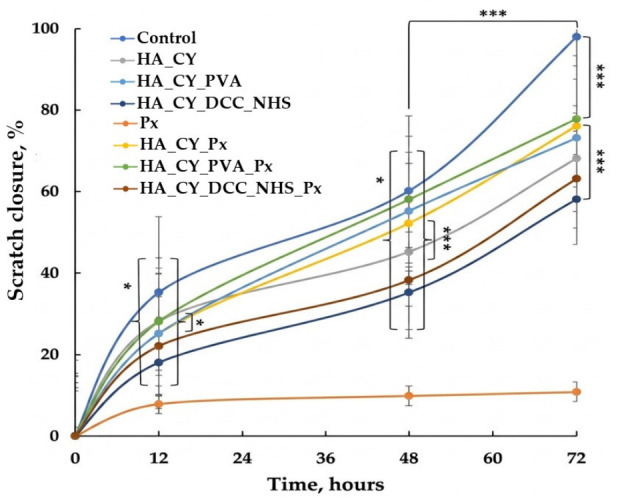
The wound closure efficiency for the tested hydrogels. Data are means ± S.D, n = 3. * *p* < 0.05, *** *p* < 0.001.

**Figure 14:**
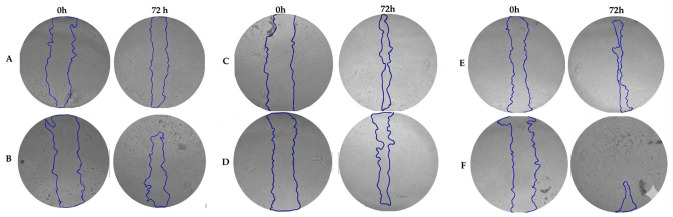
The decrease in scratch area over time. The blue overlay indicates the detected scratch area after 0 and 72 h HDFa cell migration. ((**A**)—HA_CY_DCC_NHS; (**B**)—HA_CY_DCC_NHS_Px; (**C**)—HA_CY; (**D**)—HA_CY_Px, (**E**)—HA_CY_PVA; (**F**)—HA_CY_PVA_Px). The resolution of imagine was improved with Google Gemini.

The scratch assay was performed to evaluate the influence of the developed hydrogels on in vitro fibroblast migration. The obtained results are presented in Figure 13 and Figure 14.

The wound closure profiles presented in Figure 13 revealed distinct migration behaviours depending on hydrogel composition and Px incorporation. The DMEM control group exhibited the fastest scratch closure, reaching approximately 98% closure after 72 h. In contrast, free Px solution significantly reduced cell migration, resulting in only ~11% closure at the end of the experiment. The incorporation of Px into the hydrogel matrices appeared to reduce this inhibitory effect. Although the unloaded HA_CY hydrogel exhibited slightly higher closure values during the early incubation period compared with HA_CY_Px, the Px-loaded formulation showed improved closure after 48 h, suggesting a more gradual influence of the released drug on fibroblast migration behaviour. The observed inhibitory effect of free Px may depend on the applied concentration and the specific in vitro conditions.

Among the investigated formulations, HA_CY_PVA_Px exhibited the highest wound closure efficiency among the hydrogel systems, reaching approximately 78% closure after 72 h (Figure 13 and Figure 14). This formulation showed higher closure values than both the unloaded HA_CY_PVA and the DCC/NHS-crosslinked formulations, suggesting that the combination of PVA incorporation and controlled Px release may support favourable fibroblast migration behaviour under the investigated conditions.

## 4. Discussion

The present study demonstrates how different crosslinking strategies [77,78] and polymer compositions [79] influence the physicochemical and biological performance of HA-based hydrogels. Although the individual components employed in this work have been previously reported, the comparative evaluation performed within the same formulation platform provides a clearer understanding of the structure–property relationships governing hydrogel functionality [80]. In particular, the results highlight how physical blending and covalent crosslinking distinctly modulate network organization, hydration behaviour, degradation resistance, drug release, and cellular response.

The developed hydrogels were prepared using thiolated hyaluronic acid (HA_CY) combined either with PVA through physical blending or with DCC/NHS-mediated chemical conjugation [81,82]. ATR-FTIR analysis suggested successful incorporation of the different components into the hydrogel matrices [83]. The broadening of the hydroxyl stretching region together with the slight shifts observed in thiol-associated vibrations indicated the presence of intermolecular interactions within the physically blended systems. In the chemically conjugated formulations, the appearance of more pronounced amide-associated bands suggested the formation of covalent interactions between HA and cysteine [81]. In addition, the persistence of the characteristic Px-related bands within the loaded hydrogels indicated successful drug incorporation [84,85] without evidence of major chemical degradation of the therapeutic agent [58,59].

SEM analysis revealed clear morphological differences among the formulations depending on the selected crosslinking strategy. The HA_CY hydrogels exhibited interconnected porous structures with irregular lamellar organization, while the incorporation of PVA or DCC/NHS resulted in progressively denser and more compact morphologies [77,79]. The PVA-containing systems showed stacked layered domains with reduced apparent porosity, whereas the chemically conjugated hydrogels presented more compact and less interconnected structures. Px incorporation generally preserved the overall morphology of the matrices, although slight reductions in visible porosity were observed in some formulations [84,85]. These findings indicate that both polymer composition and crosslinking chemistry strongly influence the internal architecture of the hydrogels.

The swelling behaviour further supported these structural differences. All formulations exhibited rapid initial PBS uptake followed by stabilization after approximately 4–5 h, consistent with the typical behaviour of HA-based hydrogels [10,12]. The PVA-containing formulations displayed the highest swelling degrees, which may be associated with the hydrophilic nature of PVA and the increased flexibility of the physically crosslinked networks [18,19]. In contrast, the DCC/NHS-crosslinked hydrogels showed comparatively lower swelling values, suggesting the formation of denser polymeric matrices with reduced expansion capacity [86]. Px incorporation slightly modified the swelling behaviour depending on the formulation, indicating that drug loading may also influence network hydration [30].

The differences in swelling and morphology were closely related to the drug loading and release behaviour of the hydrogels [87]. The HA_CY_PVA_Px formulation exhibited the highest encapsulation efficiency and drug loading values, suggesting improved Px retention within the denser PVA-containing matrices [79,88]. Conversely, the chemically conjugated HA_CY_DCC_NHS_Px system showed lower encapsulation efficiency, which may be associated with reduced available free volume within the compact network structure [77,89].

The release studies demonstrated that the crosslinking strategy significantly influenced Px diffusion from the hydrogels [85]. The HA_CY_Px formulation exhibited the fastest release profile, whereas the DCC/NHS-crosslinked systems showed slower and more prolonged release behaviour [88]. The PVA-containing hydrogels displayed intermediate release profiles. These findings suggest that Px diffusion was partially controlled by both network compactness and hydrogel swelling behaviour [89]. Similar relationships between crosslinking density, swelling capacity, and drug release kinetics have been previously reported for HA-based hydrogel systems [42,75].

The enzymatic degradation study further demonstrated the important role of polymer composition and crosslinking strategy in determining matrix stability [90,91]. The HA_CY formulation exhibited the highest degradation degree, indicating limited resistance toward hyaluronidase-mediated degradation. In contrast, both the PVA-containing and chemically conjugated systems exhibited improved enzymatic stability. The reduced degradation observed for these formulations may be associated with the formation of denser polymeric networks that partially restricted enzyme accessibility within the hydrated matrices. Among the investigated samples, the HA_CY_PVA_Px hydrogel exhibited the lowest degradation degree, indicating enhanced structural persistence under the investigated conditions [42,75].

Bioadhesion analysis revealed that the PVA-containing hydrogels exhibited the highest adhesive performance. This behaviour may be associated with the presence of hydrophilic hydroxyl groups [71] together with increased network flexibility and hydration, which can enhance interfacial interactions with the substrate [92,93]. In contrast, the DCC/NHS-crosslinked formulations exhibited comparatively lower adhesion values, likely due to reduced polymer chain mobility associated with the more compact network organization [70,85]. These findings indicate that crosslinking chemistry strongly affects the interfacial properties of the developed hydrogels.

The biological investigations demonstrated favourable cytocompatibility of all developed formulations toward human dermal fibroblasts. MTT assays showed that cell viability remained above 80% throughout the investigated incubation period, indicating the absence of significant cytotoxic effects [50]. Microscopy and Live/Dead staining further confirmed these observations, revealing well-spread fibroblast-like cells together with a predominance of viable cells across all formulations [72]. The obtained results suggest that neither the thiolation process nor the selected crosslinking strategies negatively affected fibroblast viability under the investigated conditions [73,74].

The scratch assay further demonstrated that hydrogel composition significantly influenced fibroblast migration behaviour [51,94]. Free Px solution markedly reduced scratch closure under the investigated conditions, whereas incorporation of Px into the hydrogel matrices appeared to reduce this inhibitory effect. Among the investigated formulations, HA_CY_PVA_Px exhibited the highest wound closure efficiency among the hydrogel systems, suggesting that the combination of PVA incorporation and controlled Px release may support favourable fibroblast migration behaviour. However, it should be noted that the scratch assay represents a simplified in vitro model that primarily reflects cell migration and partial proliferative behaviour rather than the full complexity of physiological wound healing.

Overall, the present study demonstrates that the combination of polymer composition and crosslinking strategy enables modulation of the physicochemical and biological behaviour of HA-based hydrogels. Physically crosslinked PVA-containing systems exhibited enhanced swelling, bioadhesion, and fibroblast migration, whereas chemically conjugated hydrogels showed improved structural stability and slower drug release profiles. These findings establish a formulation–property framework that may support the rational design of HA-based biomaterials tailored for specific biomedical and drug delivery applications.

### Future Directions and Translational Perspectives

The present findings provide a foundation for the continued development of HA_CY-based hydrogels for biomedical and drug delivery applications. Although the investigated formulations demonstrated favourable physicochemical properties and good in vitro cytocompatibility, additional studies are required to further optimize their functional performance and evaluate their translational potential.

Future investigations should focus on correlating hydrogel composition with mechanical behaviour, long-term stability, and degradation kinetics under physiologically relevant conditions. In particular, understanding how PVA incorporation and DCC/NHS-mediated crosslinking influence structural persistence and drug release under dynamic biological environments would support the rational design of application-specific hydrogel systems.

Further biological evaluation should also extend beyond standard cytocompatibility testing toward more comprehensive investigations involving inflammatory response, angiogenic potential, long-term cellular interactions, and in vivo performance. Animal studies will be necessary to validate the safety, biointegration, and therapeutic behaviour of the developed hydrogels under clinically relevant conditions.

From a translational perspective, the tunable swelling, adhesion, degradation, and release properties observed in this study suggest that these materials may be further explored for localized drug delivery and soft tissue engineering applications. Future work should additionally address scalability, sterilization compatibility, formulation reproducibility, and long-term storage stability in order to support potential clinical translation.

Overall, the versatility of the developed HA_CY systems highlights the importance of formulation-driven design strategies for tailoring hydrogel behaviour according to specific biomedical requirements.

## 5. Conclusions

In this study, HA-based hydrogels were successfully developed using both physical (PVA blending) and covalent (DCC/NHS-mediated) crosslinking strategies, enabling a comparative evaluation of their physicochemical and biological properties. The obtained results demonstrated that polymer composition and crosslinking chemistry significantly influenced swelling behaviour, bioadhesion, enzymatic stability, drug release, and cellular response.

The PVA-containing systems exhibited enhanced swelling capacity and improved adhesive behaviour, whereas the DCC/NHS-crosslinked hydrogels showed increased structural stability together with slower enzymatic degradation and more prolonged Px release profiles. All investigated formulations demonstrated favourable in vitro cytocompatibility toward human dermal fibroblasts, while Px-loaded hydrogels maintained controlled release behaviour and supported fibroblast migration under the investigated experimental conditions.

Overall, the present study highlights the potential of thiolated HA-based hydrogels as adaptable biomaterials whose functional behaviour can be modulated through formulation design. Although the findings are limited to in vitro investigations, the observed structure–property relationships provide a useful framework for the further development and optimization of HA-based systems for biomedical and localized drug delivery applications.

## Data Availability

The data presented in this study are available on request from the corresponding author.

## Abbreviations

The following abbreviations are used in this manuscript:

ATR-FTIR	Attenuated total reflectance Fourier transform infrared spectroscopy
Calcein-AM	Calcein-acetoxymethyl ester
CY	Cysteine
DCC	N, N′-dicyclohexylcarbodiimide
DL	Drug loading
DMEM	Dulbecco’s Modified Eagle Medium
DTNB	5,5′-dithiobis-(2-nitrobenzoic acid)
ECM	Extracellular matrix
EE	Entrapment efficiency
FBS	Fetal bovine serum
HA	Hyaluronic acid
HA-SH	Thiolated hyaluronic
HDFA	Human dermal fibroblast cells
MTT	Thiazolyl blue tetrazolium bromide
NHS	N-hydroxysuccinimide
NSAID	Nonsteroidal anti-inflammatory drug
PBS	Phosphate-buffered solution
PVA	Poly(vinyl alcohol)
Px	Piroxicam
SD	Standard deviation
SEM	Scanning electron microscope
TNB	5-thio-2-nitrobenzoic acid
